# Supplementary material for: First spikes in visual cortex enable perceptual discrimination
Source: eLife. 2018 Apr 16;7:e34044. doi: 10.7554/eLife.34044 (PMC5902162; doi:10.7554/eLife.34044)
Supplement: Supplementary file 1. — Hold time is the minimal time that the target stimulus has to spend in the reward zone for a reward to be available. Track gain is the stimulus displacement on the monitor (cm)/running distance (cm). Target probability is the fraction of stimuli that are the target stimulus (stimuli are randomly interleaved). [file elife-34044-supp1.docx]

| **MOUSE** | **STRAIN** | **EXPERIMENT** | **HOLD**  **TIME** | **TRACK GAIN** | **TARGET PROBABILITY** |  |
| --- | --- | --- | --- | --- | --- | --- |
| 1 | VGAT-ChR2 | Optogenetic silencing | 0.6 sec | 0.45 | 0.33 |  |
| 2 | VGAT-ChR2 | Optogenetic silencing | 0.9 sec | 0.6 | 0.5 |  |
| 3 | VGAT-ChR2 | Optogenetic silencing Physiology | 0.6 sec | 0.6 | 0.5 |  |
| 4 | VGAT-ChR2 | Optogenetic silencing | 1.0 sec | 0.35 | 0.33 |  |
| 5 | VGAT-ChR2 | Optogenetic silencing | 1.0 sec | 0.3 | 0.25 |  |
| 6 | VGAT-ChR2 | Optogenetic silencing | 1.0 sec | 0.6 | 0.33 |  |
| 7 | VGAT-ChR2 | Optogenetic silencing | 1.0 sec | 0.2 | 0.5 |  |
| 8 | VGAT-ChR2 | Optogenetic silencing Physiology | 0.7 sec | 0.3 | 0.5 |  |
| 9 | VGAT-ChR2 | Optogenetic silencing Physiology | 1.1 sec | 0.3 | 0.5 |  |
| 10 | WT | Physiology | 1.1 sec | 0.35 | 0.5 |  |
| 11 | WT | Physiology | 0.9 sec | 0.35 | 0.5 |  |
| 12 | WT | Physiology | 0.6 sec | 0.35 | 0.5 |  |
| 13 | WT | Physiology | 0.6 sec | 0.5 | 0.5 |  |
| 14 | WT | Physiology | 1.1 sec | 0.3 | 0.5 |  |
| 15 | WT | Cortical ablation | 1.3 sec | 0.35 | 0.5 |  |
| 16 | WT | Cortical ablation | 0.9 sec | 0.3 | 0.5 |  |
| 17 | WT | Cortical ablation | 1.3 sec | 0.3 | 0.5 |  |
| 18 | WT | Cortical ablation | 0.9 sec | 0.3 | 0.5 |  |
| 19 | WT | Cortical ablation | 0.9 sec | 0.3 | 0.5 |  |
|  |  |  |  |  |  |  |

**Supplementary File 1. Parameters for the behavioral task for each of the mice included in the main experiments.** Hold time is the minimal time that the target stimulus has to spend in the reward zone for a reward to be available. Track gain is the stimulus displacement on the monitor (cm) / running distance (cm). Target probability is the fraction of stimuli that are the target stimulus (stimuli are randomly interleaved).
